# Frequency-dependent Batesian mimicry maintains colour polymorphism in a sea snake population

**DOI:** 10.1038/s41598-022-08639-6

**Published:** 2022-03-18

**Authors:** Richard Shine, Gregory P. Brown, Claire Goiran

**Affiliations:** 1 School of Natural Sciences, Macquarie University, Sydney, NSW 2109 Australia; 2 School of Life and Environmental Sciences, University of Sydney, Sydney, NSW 2006 Australia; 3 LabEx Corail & ISEA, Université de la Nouvelle-Calédonie, BP R4, 98851 Nouméa cedex, New Caledonia

**Keywords:** Ecology, Evolution, Zoology

## Abstract

Evolutionary theory suggests that polymorphic traits can be maintained within a single population only under specific conditions, such as negative frequency-dependent selection or heterozygote advantage. Non-venomous turtle-headed sea snakes (*Emydocephalus annulatus*) living in shallow bays near Noumea in New Caledonia exhibit three colour morphs: black, black-and-white banded, and an intermediate (grey-banded) morph that darkens with age. We recorded morph frequencies during 18 consecutive years of surveys, and found that the numbers of recruits (neonates plus immigrants) belonging to each morph increased in years when that morph was unusually rare in the population, and decreased when that morph was unusually common. Thus, morph frequencies are maintained by negative frequency-dependent selection. We interpret the situation as Batesian mimicry of highly venomous sea snakes (*Aipysurus, Hydrophis, Laticauda*) that occur in the same bays, and range in colour from black-and-white banded to grey-banded. Consistent with the idea that mimicry may protect snakes from attack by large fish and sea eagles, behavioural studies have shown that smaller fish species in these bays flee from banded snakes but attack black individuals. As predicted by theory, mimetic (banded) morphs are less common than the cryptically-coloured melanic morph.

## Introduction

Polymorphic traits present a challenge to simplistic models of natural selection. Two morphs are unlikely to confer exactly the same fitness, and hence we would expect natural selection against the less-fit morph to eliminate the polymorphism^1^. An extensive literature examines why this prediction is not always met and shows that multiple morphs can be maintained by processes such as heterozygote advantage, negative frequency-dependent selection, differential habitat use based on genotype or phenotype, correlational selection, or differential selection on males and females^1–4^. Some of the most dramatic cases of polymorphism involve multiple colour-morphs that function as Batesian mimics of sympatric toxic species. In particular, some butterfly populations contain individuals with distinctive colour patterns that strongly resemble those of multiple co-occurring “models” that are avoided by visually-hunting predators^5,6^.

Although Batesian mimicry likely is widespread, most examples of this phenomenon in reptiles involve species-level resemblances between harmless mimics and toxic models, as in the coral-snake system in South America^7,8^ and the viper-colubrid system in Europe^9,10^. In contrast, studies of intraspecific colour polymorphism in reptiles mostly have focused on sex-specific morphs in lizards, maintained by asymmetric advantages in intrasexual combat among males (e.g., “rock-paper-scissors” dynamics^11^) or by sex-based differences in microhabitat use that affect the link between colour patterns and fitness^12^. The best empirical evidence for balancing selection on colour morphs in natural populations comes from longterm studies on invertebrates^13^.

Although sea snakes are diverse and abundant in tropical oceans, logistical obstacles have discouraged detailed research on these animals^14^. In the present study, we examine a species that is highly variable in colour both among and within populations: the turtle-headed sea snake *Emydocephalus annulatus*^15^. In the populations that we study in the IndoPacific archipelago of New Caledonia, most individuals are jet-black but some are brightly banded in black-and-white whereas others are intermediate^15–17^. To clarify the mechanisms that maintain this polymorphism, we analyse data from an 18-year mark-recapture program to explore the distribution of colour morphs with respect to sex and body size, and the degree to which morph frequencies remain relatively constant through time and space. If recruitment is negatively frequency-dependent, we expect that an increased proportion of snakes of a particular morph will be followed by a reduction in relative rate of recruitment of that morph, thus keeping morph frequencies stable.

## Methods

### Study site and species

We work in two small shallow (< 3 m deep) bays beside the city of Noumea, in New Caledonia (22°16′ S, 166°26′ E). The substrate is dominated by live coral, coral rubble, boulders and sand, with sea snakes primarily using coral substrates^16,18^. Water temperatures average around 27 °C in midsummer (February) and 23 °C in midwinter (August: https://www.seatemperature.org/australia-pacific/new-caledonia/noumea.htm).

Turtle-headed sea snakes (*Emydocephalus annulatus*) are medium-sized (to 800 mm snout-vent length [SVL]) hydrophiine elapids (“true” sea snakes^19^) that are entirely aquatic. Adult females produce a litter of about two large offspring (300 mm SVL) on an approximately biennial cycle^20^, usually in May–June. Courtship and mating occur primarily in the cooler months (June–August), with ovulation in spring (September–October) followed by a long gestation. Both sexes mature at about 500 mm SVL, and at two to three years of age^20^. The unusual diet of this species (eggs of demersal-spawning fishes^21^) has driven major changes in the trophic apparatus. Venom plays no role in subduing prey, resulting in a considerable reduction in size of the fangs and venom glands, potency of the venom, and ability to gape the mouth open widely. Thus, unlike almost all other sea snakes, this species is essentially non-venomous^19^.

**Figure 1:**
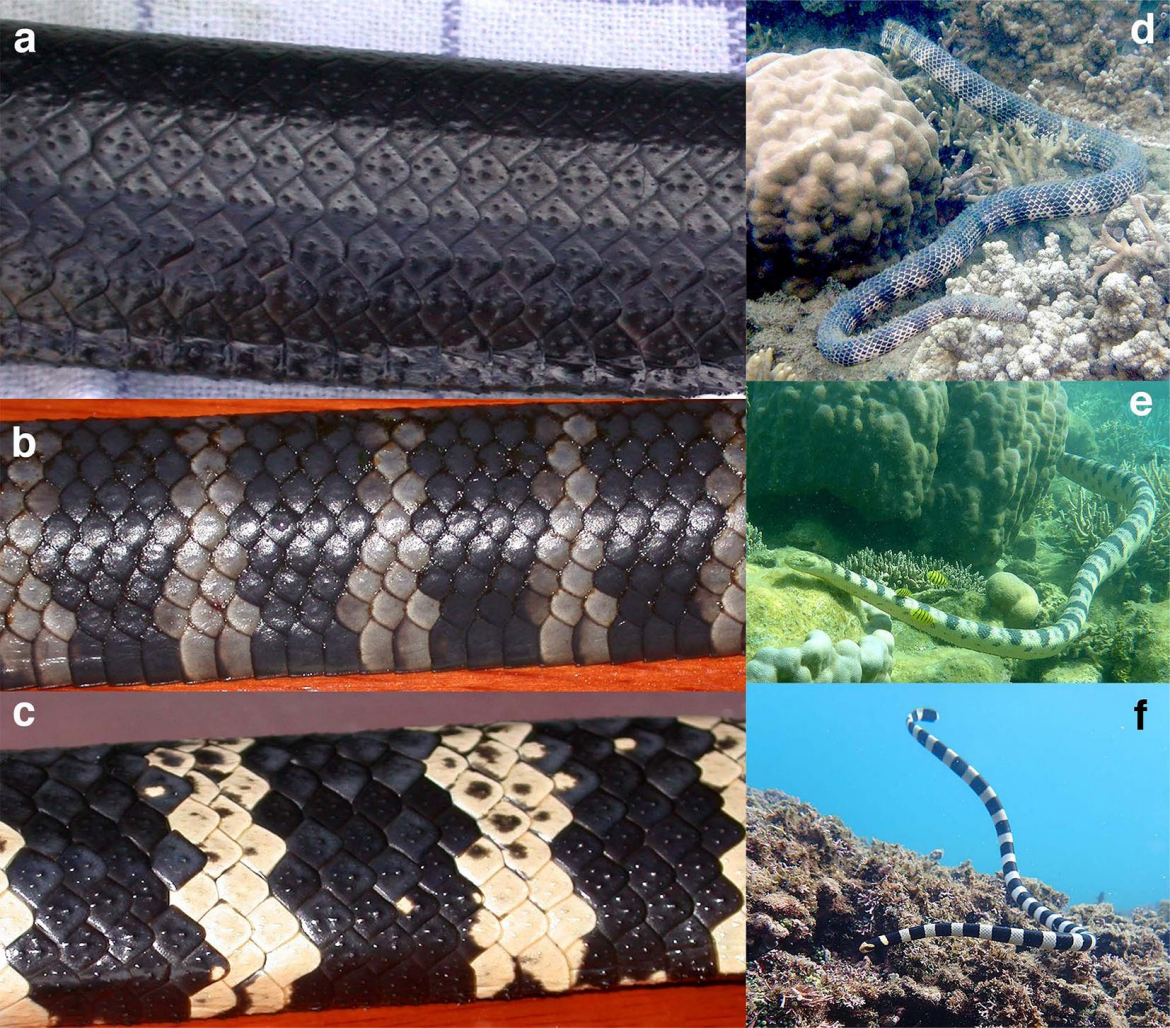
Photographs of colour morphs in turtle-headed sea snakes, *Emydocephalus annulatus*, and putative models that may be mimicked by the banded morphs. Left-hand panels show (**a**) melanic, (**b**) intermediate, and (**c**) black-and-white banded specimens of *E. annulatus*. Right-hand panels show the putative models for banded morphs (**d**) *Aipysurus duboisii*, (**e**) *Hydrophis major*, and (**f**) *Laticauda saintgironsi*. Photographs by R. Shine except for *H. major* and *L. saintgironsi* (C. Goiran).

Snakes in our study populations occur in three colour morphs: jet-black (melanic), brightly black-and-white banded, and intermediate (grey-banded: see Fig. 1). All snakes were classified to colour morph by the same observer (RS), except that CG scored snakes in 2021. To quantify inter-rater reliability, we asked 10 volunteers (colleagues and students) to classify 30 photographs of snakes into morphs. Participants were told that snakes occur in three morphs (black, black-and-white-banded, and intermediate) but were not told how many snakes fell into each category. We provided 10 randomly-selected photographs of each morph.

In previous work, we have shown that darker colours increase algal fouling (because algae preferentially settle onto dark backgrounds), increasing hydrodynamic resistance and thus, reducing swimming speeds^23^. However, black skin also confers an advantage: it facilitates elimination of trace-element pollutants from the body via sloughing, because melanin binds to those trace elements, and because of the higher sloughing frequency induced by higher rates of algal fouling^15^. Detailed surveys have not revealed any other ecological traits associated with colour morph: for example, rates of growth and survival are similar between banded and melanic individuals^23^, as is microhabitat use^16^. The mechanistic basis of colour polymorphism in *E. annulatus* is not known, but studies on other snake species have revealed a genetic basis to intraspecific colour variation, with simple Mendelian inheritance^22^.

### Surveys

Every January from 2004 to 2021, we snorkelled (in groups of 3 to 14 people) through three coral-substrate sites within two adjacent bays: the Baie des Citrons and Anse Vata (see^19^ for site details). All snakes sighted were captured by hand, and taken to a nearby laboratory for measuring, weighing and microchipping prior to release at the site of capture. Analyses using the program MARK suggest that we captured 24 to 80% of resident snakes at each site each year (mean = 54%). These slow-moving snakes are relatively easy to see, especially when they are active in shallow water, so a snake’s colour is unlikely to have affected its probability of capture.

During processing, we scored the colour of each snake into three categories (see above and Fig. 1). Pregnancy and litter sizes were assessed by palpation of the large developing embryos. We handled and scored each individual only once in each year, but many snakes were captured in multiple years. The current analysis is based on 2,673 captures of 1,427 snakes.

### Statistical analysis

We used generalized linear models implemented in JMP Pro 15.0 and SAS 9.4 to analyse data on colour morph frequencies and their correlates. We used nominal logistic regression (with a glogit link function) to assess the effects of sex body size, site, and year on the frequencies of each colour morph, using only the first capture per individual to avoid pseudoreplication. To examine variation through space and time in the proportion of snakes of each colour morph, we used models with number of individuals of each colour morph as the dependent variable, and independent variables of morph and site (to examine spatial variation) or morph and year (to examine temporal variation). Interaction terms were included and a negative binomial distribution was used to fit the model. We were unable to include both Site and Year in the same models, because not all sites were studied over the same period.

To examine survival rates for snakes of each morph, we pooled the data from Baie des Citrons south and Anse Vata (the two sites for which 17-year mark-recapture data were available) for Cormack–Jolly–Seber analysis implemented in MARK software^24^. We used colour morph as a grouping variable with three levels and ran 16 models where survival and recapture parameters were constant, varying over time, varying among colour morphs or varying interactively with time and morphs. We then compared the fit of the 16 models by ranking their corrected Akaike (AICc) values. We considered models whose AICc values differed by < 2.0 to have equivalent support.

To evaluate ontogenetic changes in colour pattern, we examined mark-recapture records to quantify the numbers of cases in which a snake remained the same colour as in earlier years, versus was classified as a different morph.

To assess frequency-dependence in recruitment, we compared the proportion of snakes of each colour morph within a given population to the proportion of snakes of that colour in the following year. We conducted this analysis using a model with study site as a factor, % of snakes in a specific colour morph as a covariate (plus the interaction between these factors), and change in % of snakes of that colour between that year and the next as the dependent variable. We ran separate analyses for each of the three colour morphs, using a Gaussian distribution.

Although proportions facilitate interpretation of patterns in the data, ratio measures can introduce statistical problems^25^. We thus conducted an alternative analysis for density-dependence that used data on actual counts: the changes in numbers of snakes of each colour morph were included as three independent variables, and the number of snakes of each colour morph in the preceding year as the dependent variable. We used a generalized mixed model for these analyses, incorporating Site as a random effect and using a Gaussian distribution.

### Ethics statement

The research was conducted under animal ethics approval 2015/880 (University of Sydney) and permit 3252-17/ARR/DENV (Province Sud, New Caledonia). All procedures involving animals were carried out in accordance with relevant guidelines and regulations (including ARRIVE guidelines).

## Results

### How discrete are the three colour morphs?

Analysis of polymorphic traits requires unambiguous definitions of each state. Our survey of inter-rater reliability gave scores from 10 observers for each of 30 snakes. Observers agreed with each other, and with our a priori classification, for 296 of those 300 evaluations (98.7%). One person scored an intermediate-phase snake as black-and-white banded, and three people scored an intermediate-phase snake as black.

**Figure 2:**
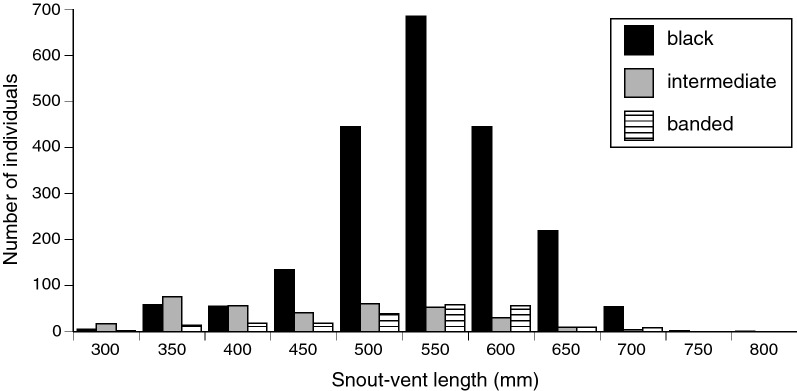
Frequency distributions of body lengths (snout-vent lengths) for each colour morph in turtle-headed sea snakes, *Emydocephalus annulatus*.

In the case of *E. annulatus*, then, the melanic and black-and-white banded states are very different (never confused with each other in the above survey), but the intermediate (grey-banded) phase is more problematic (mis-classified in 4 of 100 cases in the survey above). The three phases are very different in juvenile (young-of-the-year) individuals, but increasing melanism of intermediate-phase snakes renders them increasingly less distinguishable from melanic snakes in later age classes. That transition is evident from SVL distributions of the three morphs, with the intermediate morph most common in smaller snakes (Fig. 2). Mark-recapture records confirm that most intermediate-phase animals transitioned to darker colours through time, whereas transitions between the other morphs were uncommon (Table 1). Our analyses treat each of the three morphs as separate entities, based on the colour of the snake at the time it was captured.

**Table 1:** Ontogenetic shifts in colour in individual turtle-headed sea snakes, *Emydocephalus annulatus*, based on records of snakes captured in multiple years.

	Colour morph at first capture		
	Melanic	Intermediate	Black-and-white bands
**Colour morph at recapture**			
Melanic	901	115	4
Intermediate	40	48	6
Black-and-white bands	2	3	95

The table shows numbers of records, in some cases including multiple successive recaptures of the same individuals.

### Is colour polymorphism linked to sex, size, site or year?

Using data from first captures only (N = 1427 snakes), we found that colour-morph frequencies were similar in female and male *E. annulatus*. Combining all records, proportions were 70.4 in females versus 77.5% for males respectively for the melanic morph, 20.7 vs 14.4% for the intermediate morph, and 8.8 vs 8.1% for the black-and-white banded morph. Assessing Site effects while pooling across years, body size was the only factor that significantly affected morph frequency (F_2,1403_ = 95.46, P < 0.0001). All other main effects and interactions in the model were nonsignificant (all F < 1.66, all P > 0.16). Assessing Year effects while pooling across Sites, body size was again the only factor that significantly affected morph frequency (F_2, 1291_ = 34.58, P < 0.0001). All other main effects and interactions were nonsignificant (all F < 1.27, all P > 0.15).

### Variation in morph frequencies through space and time

Based on the total number of each morph captured at each site in each year (N = 131 data points), the three study sites were similar in terms of the proportion of individuals belonging to the melanic morph (81.8% in Baie des Citrons south vs 81.1% in Baie des Citrons north vs 74.8% in Anse Vata), intermediate morph (12.7 vs 9.5 vs 12.7%) and black-and-white-banded morph (5.6 vs 9.5 vs 15.0). A negative binomial model with colour morph and site as factors detected a marginally significant divergence among sites in the relative numbers of each morph (interaction morph*site F_4,122_ = 2.46, P = 0.049), with Anse Vata having slightly more black-and-white morphs than did the other sites.

**Figure 3:**
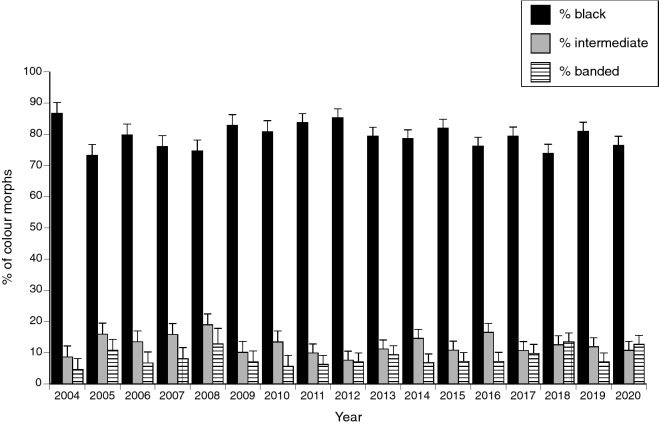
The proportion of snakes (*Emydocephalus annulatus*) belonging to each colour morph through time, based on annual surveys over an 18-year period. The graph shows mean annual values for the three study sites, with associated standard errors.

The total numbers of snakes collected per year varied across the 18 years of study (negative binomial model with year and morph as factors, year effect F_16,80_ = 3.54, P < 0.001) but with no significant change in relative numbers of the three morphs among years (interaction year*morph F_32,80_ = 0.76, P = 0.80; see Fig. 3).

### Effect of colour morph on survival

A model with time-varying survival and recapture rates best fit the capture data from the snakes captured and marked in Baie des Citrons south (N = 932) and Anse Vata (N = 1,132) between 2004 and 2020. Annual survival rates ranged from 41.7% ± 4.6 SE to 95.3% ± 10.0 SE. This model was a better fit than the top model in which survival varied among colour morphs (Table 2). Thus, there is no support for survival varying among colour morphs.

**Table 2:** Ranking of Cormack-Jolly-Seber models estimating survival (Phi) and recapture rate (p) of sea snakes in Baie des Citrons south and Anse Vata.

Model	AICc	Delta AICc	Num. Par
Phi(t) p(t) PIM	4333.2991	0	31
Phi(t) p(g) PIM	4344.0338	10.7347	19
Phi(t) p(.) PIM	4346.9987	13.6996	17
Phi(t) p(g*t) PIM	4347.6989	14.3998	63
Phi(g*t) p(t) PIM	4362.8464	29.5473	63
Phi(g*t) p(g) PIM	4370.5701	37.271	51
Phi(g) p(t) PIM	4373.3865	40.0874	19
Phi(g*t) p(.) PIM	4375.1359	41.8368	49
Phi(.) p(t) PIM	4376.5193	43.2202	17
Phi(g*t) p(g*t) PIM	4380.4512	47.1521	93
Phi(g) p(g*t) PIM	4384.8166	51.5175	51
Phi(.) p(g*t) PIM	4388.0454	54.7463	49
Phi(g) p(g) PIM	4397.5429	64.2438	6
Phi(.) p(g) PIM	4398.0889	64.7898	4
Phi(g) p(.) PIM	4400.0063	66.7072	4
Phi(.) p(.) PIM	4402.8755	69.5764	2

Models differ in whether or not survival and recapture rates are constant (.), vary over time (t), vary among colour morph groups (g) or vary interactively with time and group (g*t). The top-ranked model (one in which both survival and recapture rates varied over time) fit the data better than the top model in which survival varied among colour morphs (Phi(g*t) p(t). AICc = corrected Akaike Information Criteria.

### Negative frequency-dependence

**Figure 4:**
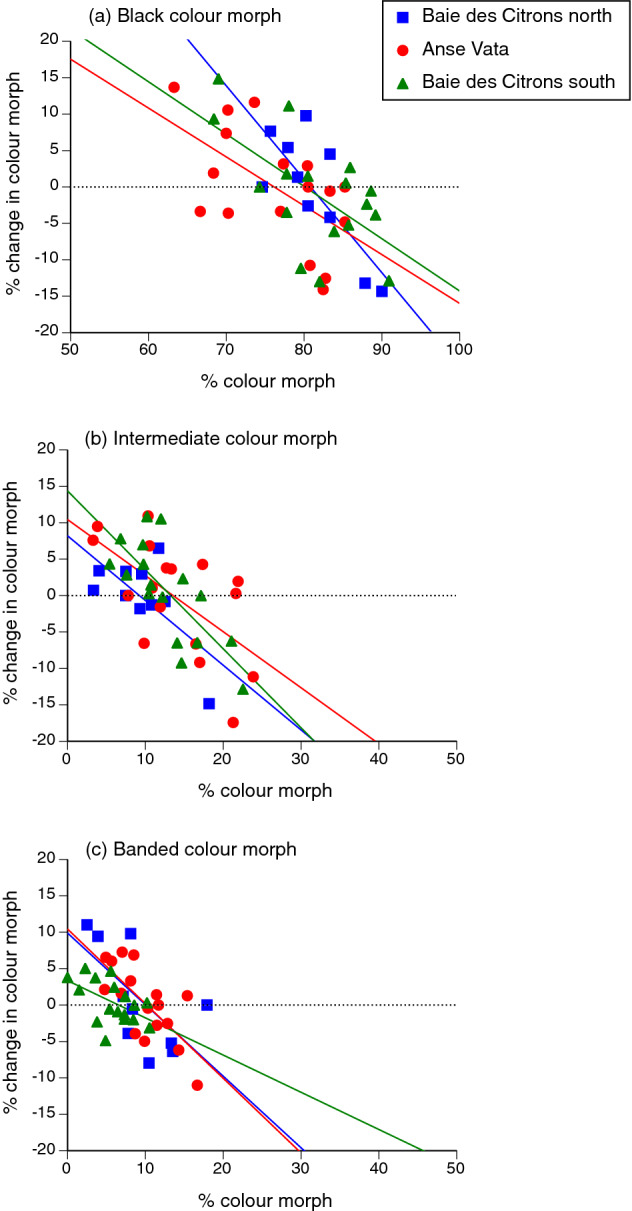
Negative frequency-dependent recruitment of turtle-headed sea snakes, *Emydocephalus annulatus*, with respect to colour morph: (**a**) black, (**b**) intermediate and (**c**) banded. The graphs show, for each colour morph, the relationship between frequency of a morph in one year (X-axis) compared to the change in that frequency during the following year (Y-axis). Regression lines are fitted to data from each of the three study sites.

Years with a higher-than-usual proportion of snakes of a particular colour morph were generally followed by lower-than-usual rates of recruitment of snakes of that morph. For all three morphs, the change in proportions was negatively related to the prior proportion of snakes exhibiting that colour morph. Negative frequency-dependence can be quantified by the main effect of the covariate (the % of the population comprising a specific colour morph in the previous year) on the % change in relative numbers of that morph from the previous year to the current one. This parameter was highly significant for the annual change in relative numbers of melanic snakes (F_1,35_ = 27.15, P < 0.0001), intermediate-phase snakes (F_1,35_ = 25.01, P < 0.0001), and black-and-white banded snakes (F_1,35_ = 38.27, P < 0.0001; see Fig. 4). We note, however, that these three tests are not statistically independent because the frequency of one morph influences the relative frequency of the others.

In an alternative analysis using data on counts rather than proportions and using Site as a random effect, the number of snakes of a given colour morph was negatively related to the change in numbers of that morph during the following year, but not to changes in numbers of other morphs. Thus, the number of banded snakes was negatively associated with the change in number of banded individuals (F_1,35.15_ = 12.31, P < 0.0015) but not with changes in the numbers of melanic or intermediate-phase snakes (respectively, F_1,35.2_ = 0.005, P = 0.95; F_1,35.14_ = 0.18, P = 0.67). Likewise, the number of melanic snakes was negatively associated with the change in number of melanic individuals (F_1,36.5_ = 4.65, P < 0.04) but not with changes in the numbers of banded or intermediate-phase snakes (F_1,35.93_ = 0.005, P = 0.95; F_1,36.1_ = 0.76, P = 0.39). Finally, the number of intermediate-phase snakes was negatively associated with the change in number of intermediate-phase individuals (F_1,35.06_ = 11.55, P < 0.002) but not with changes in the numbers of banded or melanic snakes (F_1,35.08_ = 0.18, P = 0.67 and F_1,35.21_ = 0.77, P = 0.39 respectively).

## Discussion

Independent of their sex, about 80% of the turtle-headed sea snakes in our study populations are melanic (black), but around 10% are brightly banded in black-and white. The remaining 10% exhibit medium (grey) bands when young, but darken to resemble the melanic morph as they grow older. Those proportions are similar among populations (despite very little gene flow between sites^26^) and were consistent through time across the entire study area. However, relative proportions of individuals of the three colour morphs shifted through time within each site, with negative frequency-dependence: as soon as one morph became more common than usual, recruitment of that morph declined and hence, maintained the *status quo* (Fig. 4).

What biological processes maintain numerical stability of the three colour morphs? Many of the processes identified in mathematical models, and documented in previous studies, do not apply to our study system. For example, there is no suggestion of differential selection on males and females (sex is not linked to colour), nor of ecological differences between morphs (detailed surveys have revealed no such divergences in habitat or diet^15^). Asymmetric advantages in interference competition or sexual rivalry between individuals of different morphs (as in some lizards^11^) cannot apply, because males do not engage in agonistic interactions, and do not discriminate among female colour morphs when selecting targets for courtship^27^. Heterozygote advantage also seems unlikely, because survival rates of intermediate-phase snakes (which may or may not be heterozygotes) were no higher than were those of melanic or black-and-white banded individuals.

We suggest that the stable polymorphism seen in our harmless sea snakes is due to Batesian mimicry of sympatric deadly snakes, reducing rates of predation from visually-hunting fishes and raptorial birds. Specifically, the melanic morph may be cryptic, as well as conferring advantages in eliminating pollutants from the body^15^. In contrast, resemblance in colour pattern between venomous snakes and the banded colour phases of *E. annulatus* may protect those putative mimics. In support of that hypothesis, we note:

1. annual recruitment to our study populations depends primarily on rates of survival of neonates (changes in total numbers from one year to the next are largely due to changes in the numbers of yearling snakes that survive^28^);
2. annual recruitment is higher after years when windy conditions during winter curtail predation^28^;
3. our study sites contain seven species of highly venomous sea snakes^13^ with colour patterns that resemble the putative mimetic morphs. Notably, striking black-and-white bands characterise sea kraits (*Laticauda laticaudata* and *L. saintgironsi*), and juveniles of some *Hydrophis* species (e.g., *H. major, H. coggeri*). The intermediate (grey-banded) morph of *E. annulatus* resembles *Aipysurus duboisii* and adult *H. major* (see Fig. 1 for photographs of some of these presumed mimic-model similarities). Because juvenile sea snakes tend to be more brightly coloured than conspecific adults, the strongly-banded models are similar in size to the putative mimic species, *E. annulatus*;
4. predation likely is a significant source of mortality, based on reports of sea snakes in the diets of large fishes and birds^29–34^;
5. many *E. annulatus* in our study populations have injuries to their tails, presumably the result of foraging strikes by predators (Shine et al., unpubl. data);
6. small nest-defending fishes in our study sites attack melanic snakes but ignore or avoid banded and striped snakes^35^.

Although the evidence for the Batesian mimicry hypothesis remains circumstantial, it fits well with our experience during fieldwork. Not infrequently, we have mistaken one of the banded morphs of *E. annulatus* for another snake species, and vice versa. The same mistake is evident in published work: for example, Fig. 1 of Norman et al.’s^36^ paper on mimicry ironically includes a photograph of a banded *E. annulatus* misidentified as a sea krait (*Laticauda*), the taxon both they and we have identified as a likely model for mimetic morphs. The range of colour patterns in putative models is increased by the occurrence of snakes covered with algae, rendering bands less distinct; and limited penetration of light into deeper water reduces chromatic cues^18^. Especially in deeper water, or when cloudy water and strong currents limit visibility, a harmless snake may be avoided even if it partially mimics a dangerous species.

On a broader spatial scale, the shallow inshore bays where we have studied *E. annulatus* are among a minority of places where melanism is common in this species, perhaps reflecting advantages of darker colouration in eliminating trace-element pollutants^15^. In most other areas, most individuals are banded or blotched in colour^15^, consistent with the likelihood of higher predation rates on snakes, as well as lower pollutant loads, in these less disturbed habitats.

Sea snakes may be important components of model-mimic complexes in other contexts as well. For example, some octopuses dynamically adopt a banded appearance that resembles a venomous sea snake^36^. Famously, snake-eels (e.g., *Leiuranus semicinctus, Myrichthys colubrina*) have evolved a similar banded-snake-mimicking pattern^37,38^. Dudgeon & White^39^ likewise interpret the morphology and brightly banded colours of juvenile zebra sharks (*Stegostoma fasciatum*) as mimicry of banded sea snakes. These examples suggest multiple independent evolutionary origins of banded sea-snake colouration in marine organisms, presumably because of the fitness benefits available through mimicry. In at least some cases, predators strongly avoid aposematically coloured sea-snake models^40^. Additionally, sea snakes may mimic other sea snakes. Most species of sea snakes possess potent venom and a high proportion of them are banded or blotched in colour^19^. That consistency in colour might be due to multiple processes including phylogenetic conservatism, convergent adaptation to marine habitats, and/or Mullerian mimicry. That is, deadly sea snakes may benefit from resembling other deadly species, facilitating avoidance by predators (as suggested for other types of venomous snakes^41,42^). If so, the colour polymorphism we have documented in *E. annulatus* may be part of a much broader and more complex model-mimic system.

Future work could test the interpretation of Batesian mimicry by looking into predator–prey interactions. For example, we predict that larger fishes (like the smaller ones we have already studied^35^) would direct fewer attacks towards banded morphs than towards melanic morphs, at least in a situation where banded morphs are relatively infrequent. Artificial models of snakes, differing in colour pattern, might allow an empirical test of that prediction. More generally, we need to overcome the logistical constraints of studying predator–prey interactions underwater, if we are to more robustly test the idea that colour patterns in sea snakes are driven by Batesian mimicry.

## Data Availability

Data has been deposited in the Dryad digital repository and is available at 10.5061/dryad.x3ffbg7mq.
